# Maximum salinity tolerance and osmoregulatory capabilities of European perch *Perca fluviatilis* populations originating from different salinity habitats

**DOI:** 10.1093/conphys/coz004

**Published:** 2019-02-20

**Authors:** Emil A F Christensen, Martin Grosell, John F Steffensen

**Affiliations:** 1 Marine Biological Section, Department of Biology, University of Copenhagen, Strandpromenaden 5, Helsingør, Denmark; 2 Department of Marine Biology and Ecology, Rosenstiel School of Marine and Atmospheric Science, University of Miami, 4600 Rickenbacker Causeway, Miami, FL, USA

**Keywords:** Brackish water, osmoregulation, plasticity

## Abstract

Although considered a stenohaline freshwater species, European perch (*Perca fluviatilis*) inhabit brackish waters. The present study determined the maximum salinity tolerance and osmoregulatory capability on individuals originating from brackish water and from freshwater populations. The fish were acclimated for 3 weeks to salinities of 0, 10, 12.5, 15, 17.5 and 20 after an initial stepwise increase to the target salinity. The maximum salinity tolerance was determined as the test salinity below which the fish could not acclimate and lost equilibrium. Blood plasma osmolality was measured if the fish had not lost equilibrium after the acclimation period. The maximum salinity tolerance was 17.5 for brackish water European perch and 10 for fresh water European perch. The high salinity tolerance of the brackish water European perch was caused by their ability to both hyper- and hypo-osmoregulate, whereas the freshwater originating fish could only hyper-osmoregulate. The results showed that maximum salinity tolerances and osmoregulatory capabilities depends on the origin habitat salinity. Due to genetic differentiation between European perch populations in brackish and fresh water, the possibility of brackish water European perch being a subspecies of European perch is discussed, yet vital knowledge concerning heritability of salinity tolerance traits is still missing. Regardless of species status, within-species plasticity in the ability to cope with varying salinities have substantial ecological and conservation implications and underlines the need for managing brackish water and freshwater European perch stocks separately.

## Teaser

Brackish water European perch tolerates significantly higher salinities than freshwater conspecifics due to a physiological specialization. Therefore, brackish water European perch populations may not receive recruitment from freshwater, which raises conservation issues regarding brackish water perch populations due to climate change and fisheries.

## Introduction

Environmental salinity constitutes a physiological challenge in teleosts due to osmotic water movement and ion diffusion between the environment and the internal *milieu* of the fish, predominantly occurring over the gills, which are permeable to facilitate respiratory gas exchange, acid-base regulation, and ammonia excretion (Evans *et al.*, 2005). Teleosts must keep their internal osmolalities around 300–400 mOsm kg^−1^ to maintain homeostasis (Evans *et al.*, 2005), and only tolerate a slight variation in internal osmolality (Lutz, 1972). To counteract osmotic distress, they osmoregulate (Larsen *et al.*, 2014). Osmoregulation in water with salinities below iso-osmotic level (hyper-osmoregulation) consists of producing diluted urine via the renal system and taking up ions through specialized branchial cells (Larsen *et al.*, 2014). Osmoregulation in water above iso-osmotic level (hypo-osmoregulation) is a vastly different physiological process than hyper-osmoregulation, and is obtained by imbibing ambient water, taking up the water through the gastro-intestinal tract, and excreting excess monovalent ions through specialized branchial cells and renal excretion of divalent ions (Larsen *et al.*, 2014).

The vast majority of fish species are physiologically specialized to either hyper- or hypo-osmoregulate, and have limited abilities to do both (Evans, 1984). Therefore, many species have only limited dispersal potential in brackish water of estuaries and coastal areas (Potter *et al.*, 2015). Amongst these is the European perch (*Perca fluviatilis*), a species widely distributed all over the Eurasian continent where it is common in estuaries such as the Baltic Sea (Thorpe, 1977; Craig, 2000; Couture and Pyle, 2015). There is currently inconsistency in the literature concerning the maximum salinity tolerance and osmoregulatory capability of the species. In Christensen *et al.* (2017), European perch survived at 420 mOsm kg^−1^, equivalent to a salinity of 15, whereas in Lutz (1972) and Overton *et al.* (2008) the fish succumbed at salinities above iso-osmotic conditions (ca. 300 mOsm kg^−1^, equivalent to a salinity of 10). The discrepancy may derive from intraspecific differences in salinity tolerance and osmoregulatory capability arising from whether the fish originates from varying salinities in brackish water estuaries or stable salinity habitats of freshwater lakes and streams. Population genetic studies have shown substantial differentiation among freshwater and brackish water European perch, where the origin habitat salinity explain around 20 % (Christensen *et al.*, 2016; Nesbø *et al.*, 1999; Skovrind, 2015; Skovrind et al., unpublished data). However, the discrepancy in maximum salinity tolerance could also derive from different methodological approaches between studies, and the hypothesis that origin habitat salinity determines the maximum salinity tolerance and osmoregulatory capabilities, therefore, ought to be tested within the same experimental framework.

European perch is an ecological key species, and socio-economically important for human consumption, for commercial, and for recreational fisheries (Thorpe, 1977; Craig, 2000; Couture and Pyle, 2015), and elucidating any discrepancies in maximum salinity tolerance amongst European perch populations would therefore be valuable knowledge for conservation of the species. The present study determined the maximum salinity tolerance and evaluated the osmoregulatory capability of European perch originating from brackish water and freshwater populations.

## Materials and methods

Animal care and experimental protocols followed the guidelines of the Danish Experimental Animal Inspectorate.

### Experimental animals

The brackish water European perch were obtained from a harbor site in Køge Bugt (55°31″N, 12°19E) in the western Baltic Sea where the salinity is on average 12 all year round (Skovrind *et al.*, 2013; Christensen *et al.*, unpublished data). The salinity in this area fluctuates considerably between around 0 to just above 20 during periods of sea water intrusion from Kattegat (Christensen *et al.*, unpublished data), and the change in salinity can happen with up to 10 per day. The freshwater European perch were obtained from Lake Esrom (55°58′N, 12°22E), an inland lake with no downstream connection to a brackish water European perch population. The experimental animals were caught in April, May, and June, in 2016, 2017 and 2018, by angling and with cast net and transported to the Marine Biological Section, Elsinore, Denmark (permit number 12-7410-000008) (see Table 1 for details about size and sample size). All fish were kept in freshwater (non-chlorinated Elsinore tap water) at 20°C in 60 L aerated aquaria for 3 weeks before the experiments began, to allow for acclimation to the laboratory facilities, and to alleviate any effect of former temperature acclimation. The lighting scheme was 12 h light, 12 h dark. They were fed sliced herring (*Clupea harengus*) three times a week. Excess food was siphoned from the tanks and 2/3 of the water exchanged once a week. The water was filtered continuously through biofilters.

**Table 1:** Maximum salinity tolerance of European perch (*Perca fluviatilis*) originating from brackish water (BW) and fresh water (FW).

Origin	Salinity	Amb Osm	*N*	BM	SL	Long-term acclimated?
BW	0	52	7	36.6 ± 3.8	13.4 ± 0.4	Yes
	10	300	5	51.6 ± 4.9	14.1 ± 0.3	Yes
	12.5	363	7	10.0 ± 1.5	8.5 ± 0.3	Yes
	15	428	6	11.9 ± 3.8	8.4 ± 0.8	Yes
	17.5	498	7	25.3 ± 10.8	10.9 ± 0.9	Yes
	17.5	498	7	22.0 ± 10.8	10.4 ± 1.4	No (LOE)
	20	559	10	12.3 ± 1.6	8.7 ± 0.4	No (LOE)
FW	0	51	9	16.6 ± 2.8	11.0 ± 0.9	Yes
	10	278	8	16 ± 0.9	10.3 ± 0.1	Yes
	12.5	375	6	36.3 ± 6.5	13.0 ± 0.7	No (LOE)

Ambient salinity as salinity and osmolality (Amb Osm; mOsm kg^−1^), sample size (N), body mass (BM; g), standard length (SL; cm), and whether the fish accomplished long term acclimation to the salinity (3 weeks) is given. Loss of equilibrium (LOE) is indicated.

### Maximum salinity tolerance

Brackish water and freshwater European perch were acclimated to salinities of 0, 10, 12.5, 15, 17.5 and 20 to determine their maximum salinity tolerance and osmoregulatory abilities. These salinities mimic the naturally occurring salinities in the western Baltic Sea where the brackish water European perch were obtained from. The target salinity was reached by increasing the salinity once a day starting from freshwater in the sequence 0, 10, 12.5, 15, 17.5 and 20, by adding filtered sea water from Kattegat. The fish were monitored twice a day, and euthanized if loss of equilibrium (LOE) occurred. LOE was defined as the point where the fish could not maintain an upright position, did not react to light tapping against the aquarium, and did not respond to being pinched in the tail. After 3 weeks at the target salinity, a blood sample was taken by caudal puncture. The blood sample was centrifuged (Sprout, Heathtrow Scientific, IL, USA) at 2000 *g* for 3 min, and the plasma osmolality measured in an osmometer (Vapor Pressure Osmometer 5520, Wescor Environmental, Logan, UT, USA), calibrated with the manufacturer’s standards. The fish were fed throughout the acclimation period, yet fasted 3 days prior to blood sampling. The maximum salinity tolerance was defined as the salinity level below which all fish reached LOE within 3 weeks.

### Data analyses

The blood plasma osmolality values at each treatment were tested for normality with Shapiro–Wilk’s tests, and for variance homogeneity within each of the populations with Levene’s tests. The blood plasma osmolalities at the different salinities was compared within each population with one-way ANOVAs, followed by Tukey’s post hoc test if normally distributed and the variances homogeneous, and with one-way ANOVAs with Welch correction, followed by Games-Howell post hoc test, if normally distributed and the variances heterogeneous. All statistics were computed in SPSS statistics 25 (IBM, Armonk, NY, USA), and the significance level set to an *α* value of 0.05.

## Results

**Figure 1:**
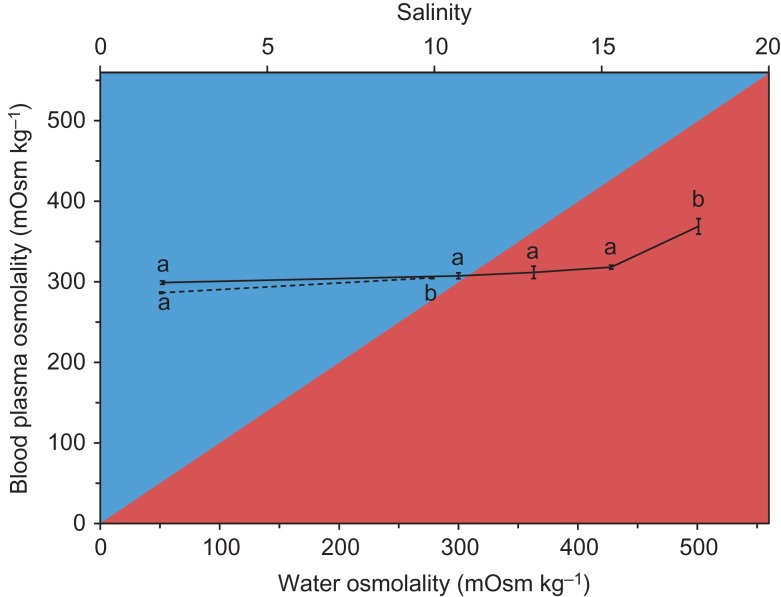
Osmoregulatory capabilities of European perch (*Perca fluviatilis*). Blood plasma osmolality is shown in relation to ambient water osmolality. The black line represents fish of brackish water origin, the dashed line represents fish of freshwater origin. Data is shown as the average ± SE. Different letters are assigned to significantly different groups within each population (Games-Howell tests used for the brackish water European perch, Tukey’s test used for the freshwater European perch). For details about sample sizes, please consult Table 1. The blue area is where the fish hyper-osmoregulate and the red area is where the fish hypo-osmoregulate (osmoregulation in water with an osmolality lower and higher than the internal osmolality of the fish, respectively).

The maximum salinity tolerance of the brackish water European perch was 17.5, yet half of individuals reached LOE before 3 weeks at this salinity (Table 1). The brackish water European perch attempted acclimated to a salinity of 20 reached LOE on a median time of 6 days. The maximum salinity tolerance of the freshwater European perch was 10, and the fish reached LOE on a median time of 10 days at a salinity of 12.5. The blood plasma osmolality ranged from 286 to 369 mOsm kg^−1^, and increased significantly approaching the maximum salinity tolerance for both the brackish water European perch and the freshwater European perch [one-way ANOVA with Welch correction, *F*(4, 12.692) = 15.39, *P* < 0.001, and one-way ANOVA, *F*(1,15) = 5.931, *P* = 0.028, respectively] (Fig. 1). The brackish water European perch were able to both hyper- and hypo-osmoregulate, whereas the freshwater European perch were only able to hyper-osmoregulate.

## Discussion

### Maximum salinity tolerance and osmoregulatory capability

The brackish water European perch had a maximum salinity tolerance of 17.5, which was substantially higher than the maximum salinity tolerance of the freshwater European perch (10). Intriguingly, the brackish water European perch had the ability to both hyper- and hypo-osmoregulate, in contrast to the freshwater European perch, which could only hyper-osmoregulate. Other freshwater European perch populations have also been shown unable to hypo-osmoregulate (Lutz, 1972; Overton *et al.*, 2008), which must therefore be considered normal amongst freshwater European perch. Hyper- and hypo-osmoregulation in teleosts are two fundamentally different physiological processes (Larsen *et al.*, 2014), and the ability of the brackish water European perch to do both must thus be associated with physiological specialization to life in brackish water. This, in turn, increases the maximum salinity tolerance and thus enhances the species distribution potential in brackish water.

### 

Tagging studies alongside physio-chemical measurements have shown that brackish water European perch in the western Baltic Sea experience salinities around 12 throughout the year, ranging from 0 to above 20 (Olsen, 2002; Skovrind *et al.*, 2013; Christensen *et al.*, unpublished data). These populations conduct winter migrations into lower reaches of streams (Olsen, 2002; Skovrind *et al.*, 2013; Christensen *et al.*, unpublished data), presumably to save energy on osmoregulation at low temperatures (Christensen *et al.*, 2017). Spawning also occurs in brackish water with successful hatching (Christensen *et al.*, 2016; Skovrind *et al.*, 2013) at salinities higher than the tolerance salinities of eggs and fry in other European perch populations (Klinkhardt and Winkler, 1989; Tibblin *et al.*, 2012). Exposure to high environmental salinities throughout the whole life cycle of European perch in the western Baltic Sea likely adds a substantial selection pressure for higher salinity tolerance in brackish water European perch populations.

### Is brackish water European perch an independent species?

Varying salinity tolerances and osmoregulatory capability can be determining factors when assessing management units of closely related fish populations, even to the point of classifying differentiating populations as subspecies or sister species. For instance, in whitefish (*Coregonus* spp.), a population endemic to the Wadden Sea has been classified as North Sea houting (*Coregonus oxyrhynchus*), an independent species, in part due to its anadromous lifestyle and higher salinity tolerance compared to European whitefish (*Coregonus lavaretus*) (Hansen *et al.*, 1999). In pupfish (*Cyprinodon variegatus*), a population endemic to central Florida is designated a subspecies, the Lake Eustis pupfish (*Cyprinodon variegatus variegatus*), on behalf of its distinct osmoregulatory capability, and debate is ongoing as to whether it should be regarded an independent species (Brix and Grosell, 2013).

Nesbø *et al.* (1999) demonstrated genetic differentiation among stationary and anadromous European perch populations in the northern Baltic region. Furthermore, Skovrind (2015) showed significant genetic differentiation between European perch from fresh water and brackish water in the western Baltic region, using full genome representative sequencing on six freshwater, and six brackish water populations (*N* = 190). The two European perch populations of the present study are from the same study sites as the ones used in Skovrind (2015), and it is likely that the population structure and genetic differentiation is associated with differences in maximum salinity tolerance and osmoregulatory capability between brackish water and fresh water European perch populations. Together with the genetic differentiation, these physiological differences in relation to origin habitat salinity could indicate an emerging speciation between brackish water and freshwater European perch, as it is argued for North Sea houting and Lake Eustis pupfish (Hansen *et al.*, 1999; Brix and Grosell, 2013). However, it remains untested to what extend salinity tolerance and osmoregulatory capability is an inheritable characteristic, which must be clear before a separate species status may apply.

### Conservation perspectives

Regardless of species status, the results of the present study are valuable information for ecologists and conservation biologists. Locally, environmental salinity in estuaries and coastal areas is currently susceptible to changes due to altering patterns in river-runoff and evaporation associate with climate change (Harley *et al.*, 2006; Vuorinen *et al.*, 2015). Furthermore, substantial recreational and commercial fisheries for European perch take place in the Baltic Sea (Craig, 2000; Thorpe, 1977; Christensen *et al.*, unpublished data). To conserve the species, and mediate ecological effects of climate change in these areas, the increased salinity tolerance and osmoregulatory capability of brackish water European perch needs to be recognized, as it is unlikely that a depleted stock will receive successful recruitment from nearby freshwater stocks.

It remains unknown whether the varying salinity tolerance and osmoregulatory capability applies to other species of freshwater fishes in estuaries and coastal regions (Potter *et al.*, 2015), or is unique to European perch in the western Baltic Sea. Further exploration into this matter could be target for future studies.
